# Health Outcomes Following Engagement With a Digital Health Tool Among People With Prediabetes and Type 2 Diabetes: Prospective Evaluation Study

**DOI:** 10.2196/47224

**Published:** 2023-12-28

**Authors:** Farah Abdelhameed, Eilish Pearson, Nick Parsons, Thomas M Barber, Arjun Panesar, Charlotte Summers, Michaela de la Fosse, Petra Hanson

**Affiliations:** 1 Warwickshire Institute for the Study of Diabetes, Endocrinology and Metabolism University Hospitals Coventry and Warwickshire NHS Trust Coventry United Kingdom; 2 Warwick Medical School University of Warwick Coventry United Kingdom; 3 Statistics and Epidemiology Unit, Warwick Medical School University of Warwick Coventry United Kingdom; 4 Diabetes Digital Media Health Coventry United Kingdom

**Keywords:** application, diabetes, digital health tool, digital health, eHealth, mHealth, mobile app, mobile health, prediabetes, quality of life, type 2 diabetes

## Abstract

**Background:**

Diabetes is a worldwide chronic condition causing morbidity and mortality, with a growing economic burden on health care systems. Complications from poorly controlled diabetes are associated with increased socioeconomic costs and reduced quality of life. Smartphones have become an influential platform, providing feasible tools such as health apps to deliver tailored support to enhance the ability of patients with diabetes for self-management. Gro Health is a National Health Service division X–certified digital health tool used to deliver educational and monitoring support to facilitate the development of skills and practices for maintaining good health.

**Objective:**

This study aims to assess self-reported outcomes of the Gro Health app among users with diabetes and prediabetes and identify the factors that determine engagement with the digital health tool.

**Methods:**

This was a service evaluation of self-reported data collected prospectively by the developers of the Gro Health app. The EQ-5D questionnaire is a standardized tool used to measure health status for clinical and economic appraisal. Gro Health users completed the EQ-5D at baseline and 6 months after using the app. Users provided informed consent for the use of their anonymized data for research purposes. EQ-5D index scores and visual analogue scale (VAS) scores were calculated at baseline and 6 months for individuals with prediabetes and type 2 diabetes. Descriptive statistics and multiple-regression models were used to assess changes in the outcome measures and determine factors that affected engagement with the digital tool.

**Results:**

A total of 84% (1767/2114) of Gro Health participants completed EQ-5D at baseline and 6 months. EQ-5D index scores are average values that reflect people’s preferences about their health state (1=full health and 0=moribund). There was a significant and clinically meaningful increase in mean EQ-5D index scores among app users between baseline (0.746, SD 0.23) and follow-up (0.792, SD 0.22; *P*<.001). The greatest change was observed in the mean VAS score, with a percentage change of 18.3% improvement (61.7, SD 18.1 at baseline; 73.0, SD 18.8 at follow-up; *P*<.001). Baseline EQ-5D index scores, age, and completion of educational modules were associated with significant changes in the follow-up EQ-5D index scores, with baseline EQ-5D index scores, race and ethnicity, and completion of educational modules being significantly associated with app engagement (*P*<.001).

**Conclusions:**

This study provides evidence of a significant positive effect on self-reported quality of life among people living with type 2 diabetes engaging with a digital health intervention. The improvements, as demonstrated by the EQ-5D questionnaire, are facilitated through access to education and monitoring support tools within the app. This provides an opportunity for health care professionals to incorporate National Health Service–certified digital tools, such as Gro Health, as part of the holistic management of people living with diabetes.

## Introduction

Type 2 diabetes (T2D) is a chronic condition that is a leading cause of morbidity and mortality worldwide [1]. It is an emerging public health crisis with a growing clinical, social, and economic burden on both patients and health care systems. Worldwide, there are around 462 million individuals affected by T2D, and the numbers are dramatically rising in every country [2]. In the United Kingdom, 1 in 14 people is estimated to have diabetes, with type 2 accounting for 90% of cases [3]. Complications arising from poorly controlled diabetes include coronary heart disease, kidney disease, along with retinopathy and neuropathy, which can lead to blindness and limb amputation, respectively [4]. These systemic complications have been associated with both increased socioeconomic costs and a reduced quality of life. In a landmark UK-based study, the cost for T2D was estimated to be £8.8 billion (US $11 billion) and £13 billion (US $17 billion) in direct and indirect costs [5]. The burden of diabetic complications has not only translated to worsening health outcomes with increased risk of mortality but also to a lower health-related quality of life (HRQoL) [6-8]. The global burden of disease study identified diabetes as one of the top 10 causes of reduced life expectancy and demonstrated that high fasting glucose level was the third most common risk factor for disability-adjusted life years [9].

The treatment of T2D aims to establish control over blood glucose to reduce the associated risk of complications and disability. In addition to medical management, this can also be achieved through lifestyle modifications, including healthy eating, physical activity, and regular blood sugar monitoring, among others. It is well established in the literature that lifestyle interventions are capable of yielding significant clinical improvements, including remission, in patients with diabetes, as demonstrated in a 2021 study in the Netherlands [10,11]. However, these lifestyle modifications often depend on the development of skills and practices that aim to facilitate self-management. The term self-management refers to the responsibility given to patients to adhere to good health practices to enable them to monitor and manage their own disease outside clinical settings [12,13]. Research has shown that education and self-management play a crucial role in helping people living with diabetes achieve metabolic control [14,15], which consequently reduces their risk of developing complications and eases the burden on health care systems by encouraging patient autonomy. This highlights the importance of developing technologies to facilitate self-care and the achievement of therapeutic goals for people living with diabetes [16].

Smartphones have become an influential platform, providing digital tools (often in the form of an app) to deliver tailored support and support self-management [16,17]. There is a growing body of evidence to support the use of health apps as successful adjuvants in diabetes management, which has yielded clinically significant metabolic improvements. A 2010 meta-analysis showed that there was strong evidence supporting the notion that the use of mobile app interventions can lead to significant improvements in glycemic control among patients with T2D [17]. This finding was further reinforced in a systematic review by Bonoto et al [18], which also confirmed the efficacy of mobile apps in the management of patients with diabetes.

In addition to clinical benefits, digital tools can improve an individual’s quality of life by providing mental health support and personalized coaching, leading to greater confidence to manage day-to-day life [19,20]. Improved quality of life has been long regarded as a fundamental goal for all diabetic management interventions, with HRQoL measures being used in the evaluation of health care interventions, including cost-effectiveness [21]. The EQ-5D-5L questionnaire is a standardized HRQoL tool used to measure health status for clinical and economic appraisal [22]. The EQ-5D allows for health states to be reported as calculated index values, which can be used for economic evaluation [22,23]. These health index scores reflect how good or bad a health state is according to the preferences of the population within a certain country [22].

In this study, we assess the impact of engagement with a digital behavioral change app (Gro Health) that is certified by the National Health Service (NHS) on the self-reported health outcomes among people diagnosed with T2D or prediabetes. The EQ-5D questionnaire was used to capture outcomes as it is a widely recognized and validated tool. We also aim to identify the factors that determine engagement with digital technologies and posit that greater use of the digital platform will lead to improvements in HRQoL.

## Methods

### Intervention

Gro Health is an NHS-certified digital health tool used to deliver health prevention, chronic disease management, home monitoring, and elective care support for people waiting for treatment before treatment and posttreatment rehabilitation. A clinical dashboard enables clinical teams to remotely assess user engagement with the app and monitor patients’ health. The platform itself provides personalized education and behavioral change program streams developed in conjunction with NHS clinical teams specifically for people living with T2D, prediabetes, gestational diabetes, obesity, nonalcoholic fatty liver disease, polycystic ovarian syndrome, hypertension, high cholesterol, or cardiac rehabilitation (Multimedia Appendix 1). Gro Health is the highest Organisation for the Review of Care and Health Apps (ORCHA)–rated health app (96%), assessed on user experience, data assurance, and clinical validation [24]. The Gro Health platform facilitates personalized digital health by providing evidence-based structured education, guided behavioral change activities, weekly digital meetups and community support, health tracking, and data-driven insights to users based on their data collected on signup and as it changes through use of the platform. Gro Health uses the capability, opportunity, motivation, and behavior model of behavior change, which identifies 3 factors (capability, opportunity, and motivation) that need to be present for any behavior to occur [25]. These factors interact over time, so that behavior is seen as part of a dynamic system with positive and negative feedback loops. A recently reported study demonstrated that users (general population, rather than people living with T2D) of Gro Health had improvements in symptoms of stress, anxiety, and depression measured through standardized questionnaires over 12 weeks [25].

### Ethical Considerations

This was a service evaluation of self-reported data collected prospectively by the developers of the Gro Health digital tool, Diabetes Digital Media. Participants were not paid for their participation, and they accessed the Gro Health app for free as part of their NHS care. Participants downloaded the app and agreed to the terms of service and privacy policy of the Gro Health app, which included informed consent to use their anonymized data for research purposes. Minimal deidentified user data required for the analyses were collected. The local hospital research and development department was contacted and confirmed that no registration was necessary. This was an analysis of already collected data that users consented to sharing for research purposes.

### Participant Selection

The Gro Health app was offered to people, aged 18 years or older with a confirmed diagnosis of T2D or prediabetes, who presented for any reason between January and August 2021 at 13 NHS primary care settings in England, as part of their clinical care if the consulting health care professional felt it was appropriate. People who accepted signposting were given a Gro Health referral card or emailed a digital referral code, which was redeemed on the app or website. Those who did not have a diagnosis of prediabetes or T2D were not offered the Gro Health app. However, since the diagnosis of T2D or prediabetes was predetermined in primary care, we had no means of verifying that each participant included in this study met the clinical criteria for diagnosis as the data were anonymized. Of 2114 registrations from NHS primary care setting referrals, 1767 Gro Health study participants completed the EQ-5D at baseline and 6 months after registering on the app (1767/2114, 83.6%).

### Study Measures

Upon study sign-up (baseline), participants were asked to report their age, sex, health goal, race and ethnicity, monthly income, and diagnosis of any preexisting health conditions. They were also asked to complete the EQ-5D questionnaire. At 6 months, participants were asked to complete the same scale again in the same format. User engagement with the Gro Health app was monitored and recorded as completion of the education program tailored for patients with prediabetes and T2D, respectively.

Assessment of HRQoL was undertaken using the EQ-5D questionnaire. The EQ-5D involves self-reporting of health status in terms of 5 dimensions, including mobility, self-care, usual activities, pain, and anxiety or depression (Multimedia Appendix 2). Each dimension is rated on a 1-5 severity scale. Responses from these dimensions are then coded for each patient and converted into a single-weighted index score using population preference scores. In this study, we used the EQ-5D-5L value set for England to derive the index scores [23]. These EQ-5D index scores reflect how good or bad a health state is according to the preferences of the population within a certain country. A range of –0.594 to 1 can be obtained for EQ-5D index scores, where a value of 1 represents perfect health, 0 represents a health state equivalent to death, and a score of less than 0 represents a state worse than death. User data on EQ-5D index scores and visual analogue scale (VAS) scores were calculated from the EQ-5D responses at baseline and 6-month follow-up for all users included in the study.

### Statistical Analysis

Descriptive statistics were used to summarize the socioeconomic demographics and clinical characteristics of participants. Percentages were used to summarize categorical variables and continuous variables, were summarized by the mean and SD according to the published user guide by EuroQol for analyzing EQ-5D-5L data [22]. Differences between socioeconomic demographic and clinical factors on self-reported HRQoL outcomes (eg, EQ-5D index scores and VAS scores) were evaluated using 2-tailed *t* tests for continuous variables and chi-square tests for categorical variables. Stepwise multiple linear regression modeling was used to evaluate the impact of predictor variables such as age, sex, race and ethnicity, income, engagement with education, baseline EQ-5D index scores on the outcome variables, EQ-5D index scores at the follow-up, and time spent on the app, which was a surrogate marker for engagement with the app. A *P* value of <.05 was considered statistically significant. All statistical analyses were carried out in SPSS software (version 27.0; IBM Corp).

## Results

### Overview

Of 2114 registrations from NHS primary care setting referrals, 1767 Gro Health participants completed the EQ-5D at baseline and 6 months after registering on the app (1767/2114, 83.6%). App engagement was measured through total minutes of use, an analytic indicator used in previous studies to evaluate the effective engagement of digital health apps [25]. The mean number of engaged minutes with the Gro Health app was 268 (SD 98.3) minutes, as recorded during the 6-month study period.

### Baseline Sociodemographic Characteristics of Participants

Table 1 summarizes the baseline socioeconomic and clinical characteristics of the participants. Among 1767 users, 1129 (63.8%) were female, mean age was 49 (SD 12.7) years, 1536 (86.9%) were White, and 840 (47.5%) had an income of more than £21,000 (US $26,600). Regarding their clinical status, 76.7% (1355/1767) had T2D and the rest had prediabetes at the time they signed up for the app.

**Table 1:** Baseline demographic and clinical characteristics (n=1767). Not all users agreed to share their data, and hence the total numbers may not sum up to 1767.

Characteristic			Values
Age (years), mean (SD)			49.2 (12.7)
**Sex, n (%)**			
	Female	1129 (63.8)	
	Male	616 (34.8)	
**Race and ethnicity, n (%)**			
	White	1536 (86.9)	
	Southeast Asian	105 (5.9)	
	Black	46 (2.6)	
	East Asian	13 (0.7)	
	Multiracial	40 (2.3)	
**Income (£1=US $1.3), n (%)**			
	<£13,000	234 (13.2)	
	£13,000-£20,999	220 (12.5)	
	£21,000-£25,999	163 (9.2)	
	£26,000-£31,999	144 (8.1)	
	£32,000-£39,999	153 (8.7)	
	£40,000-£49,999	122 (6.9)	
	>£50,000	258 (14.6)	
Time spent on the app (minutes), mean (SD)			268 (98.3)
**Educational program completed, n (%)**			
	Yes	896 (50.7)	
	No	560 (31.7)	
**Clinical status, n (%)**			
	Type 2 diabetes	1355 (76.7)	
	Prediabetes	412 (23.3)	

### Changes in Health-Related Quality of Life (EQ-5D Questionnaire)

Participants’ responses over 5 levels in each of the 5 domains of the EQ-5D questionnaire at baseline and at 6-month follow-up are shown in Table 2. Of the 1767 participants, no problems at all at baseline in mobility, usual activities, self-care, pain, and anxiety or depression were reported by 1073 (60.7%), 1127 (63.8%), 1538 (87%), 665 (37.6%), and 847 (47.9%) participants, respectively. No significant differences in the reported outcome of the individual EQ-5D domains were noted at follow-up, as seen in Table 2 (chi-square test: *P*=.98 for mobility, *P*=.93 for self-care, *P*=.94 for activity, *P*=.20 for pain, and *P*=.47 for anxiety or depression).

**Table 2:** Number and percentage of user responses in the 5 domains of the EQ-5D at baseline and follow-up.

		Baseline, n (%)	Follow-up, n (%)
**Mobility**			
	Unable to walk	13 (0.7)	18 (1)
	Severe problems	120 (6.8)	102 (5.8)
	Moderate problems	225 (12.7)	272 (15.4)
	Slight problems	336 (19)	281 (15.9)
	No problems	1073 (60.7)	1094 (61.9)
**Self-care**			
	Unable to wash or dress	2 (0.1)	6 (0.3)
	Severe problems	38 (2.2)	25 (1.4)
	Moderate problems	74 (4.2)	57 (3.2)
	Slight problems	115 (6.5)	81 (4.6)
	No problems	1538 (87)	1598 (90.4)
**Activity**			
	Unable to do usual activities	26 (1.5)	27 (1.5)
	Severe problems	73 (4.1)	50 (2.8)
	Moderate problems	183 (10.4)	119 (6.7)
	Slight problems	358 (20.3)	379 (21.4)
	No problems	1127 (63.8)	1192 (67.5)
**Pain**			
	Extreme pain or discomfort	36 (2)	25 (1.4)
	Severe pain or discomfort	98 (5.5)	98 (5.5)
	Moderate pain or discomfort	317 (17.9)	197 (11.1)
	Slight pain or discomfort	651 (36.8)	489 (27.7)
	No pain or discomfort	665 (37.6)	958 (54.2)
**Anxiety or depression**			
	Extremely anxious or depressed	17 (1)	8 (0.5)
	Severely anxious or depressed	73 (4.1)	30 (1.7)
	Moderately anxious or depressed	265 (15)	326 (18.4)
	Slightly anxious or depressed	565 (32)	389 (22)
	Not anxious or depressed	847 (47.9)	1014 (57.4)

Overall health state, combining all of these domains, showed that 353 out of 1767 participants (20%) reported no problems in any of the EQ-5D domains (described as perfect health); 1164 out of 1767 users (65.9%) reported problems in at least 1 domain not worse than a level 3 (described as moderate health); and the remaining 250 (14.1%) users reported problems worse than a level 3 in at least 1 domain (described as severe health; Multimedia Appendix 3).

### Changes in EQ-5D Index Scores and VAS Scores

EQ-5D index scores reflect how good or bad a health state is, and this is adjusted according to the preferences of the population within a certain country. EQ-5D index scores were calculated at baseline and at follow-up for all participants, who were all based in the United Kingdom. The mean EQ-5D index score for this cohort significantly improved from 0.746 (SD 0.23) at baseline to 0.792 (SD 0.22) at 6-month follow-up (paired *t* test: *P*<.001). VAS scores were also analyzed for participants, and these also demonstrated a significantly positive change over time (mean 61.7, SD 18.1 at baseline and mean 73.0, SD 18.8 at follow-up; *P*<.001).

Table 3 shows the association of socioeconomic and clinical factors with the mean EQ-5D index and VAS scores between baseline and follow-up. EQ-5D index scores were higher at follow-up for female individuals (paired *t* test: *P*<.001), participants of White race (*P<*.001), participants with income of more than £25,999 (US $33,000; *P=*.009), and participants with both diabetes and prediabetes (*P<*.001). Mean EQ-5D index scores were only noted to be lower at follow-up in participants of Southeast Asian ethnicity (paired *t* test: *P*=.04). In contrast, average VAS scores significantly improved for all users at follow-up, irrespective of any differences in sociodemographic factors or clinical status (*P*<.001).

**Table 3:** Mean EQ-5D index scores and visual analogue scale scores according to sex, race, ethnicity, income, and clinical status.

Scales and patient demographics					Baseline			Follow-up		
**EQ-5D index value, mean (SD)**										
	**Sex**									
		Female	0.730 (0.23)			0.789 (0.22)				
		Male	0.777 (0.24)			0.797 (0.23)				
	**Race and ethnicity**									
		White	0.737 (0.24)			0.792 (0.22)				
		Southeast Asian	0.827 (0.16)			0.773 (0.25)				
		Black or Caribbean	0.825 (0.16)			0.834 (0.16)				
		East Asian	0.821 (0.23)			0.846 (0.09)				
		Mixed	0.764 (0.20)			0.786 (0.22)				
	**Income** **(£1=US $1.3)**									
		<£13,000	0.646 (0.28)			0.765 (0.23)				
		£13,000-£20,999	0.695 (0.26)			0.785 (0.22)				
		£21,000-£25,999	0.730 (0.20)			0.790 (0.24)				
		£26,000-£31,999	0.760 (0.22)			0.777 (0.22)				
		£32,000-£39,999	0.794 (0.19)			0.806 (0.02)				
		£40,000-£49,999	0.789 (0.23)			0.819 (0.22)				
		>£50,000	0.818 (0.17)			0.805 (0.21)				
	**Clinical status**									
		Prediabetes		0.772 (0.20)			0.807 (0.21)			
		Type 2 diabetes		0.738 (0.24)			0.788 (0.23)			
**Visual Analogue Scale score, mean (SD)**										
	**Sex**									
		Female		60.9 (18.5)			72.3 (19.4)			
		Male		63.3 (17.4)			74.5 (17.7)			
	**Race and ethnicity**									
		White		61.5 (18.3)			72.9 (18.8)			
		Southeast Asian		62.5 (16.7)			72.9 (18.4)			
		Black or Caribbean		67.5 (14.4)			79.5 (15.2)			
		East Asian		62.9 (15.0)			74.5 (15.3)			
		Mixed		60.5 (20.4)			72.5 (20.8)			
	**Income** **(£1=US $1.3)**									
		<£13,000		55.9 (20.8)			67.1 (21.4)			
		£13,000-£20,999		59.4 (19.0)			70.8 (19.7)			
		£21,000-£25,999		61.2 (16.7)			72.2 (17.9)			
		£26,000-£31,999		61.4 (19.5)			72.2 (20.4)			
		£32,000-£39,999		64.7 (16.6)			75.8 (17.3)			
		£40,000-£49,999		63.6 (16.5)			75.1 (16.4)			
		>£50,000		64.8 (14.7)			75.9 (16.2)			
	**Clinical status**									
		Prediabetes		63.5 (15.7)			74.8 (16.9)			
		Type 2 diabetes		61.2 (18.8)			72.5 (19.3)			

### Engagement With the Educational Program and Predictors of EQ-5D Index Scores at Follow-Up

Time spent in the app averaged 268 (SD 98.3) minutes, with roughly half of users (896/1767, 50.7%) completing the educational component of the app. The Gro Health app includes a personalized educational program as a component for people with prediabetes and T2D, respectively. Completers of the educational program spent a mean of 282.1 (SD 86.8) minutes on the app, compared to just 241.1 (SD 110.4) minutes for users who did not complete the educational component (independent samples *t* test: *P*<.001).

The results from the stepwise multiple linear regression modeling, where the dependent variable was the follow-up EQ-5D index score, are presented in Table 4. Predictor variables evaluated in the model included baseline EQ-5D index scores, time spent on the app, age, income, engagement with the educational program, race and ethnicity, and sex. The best-fitting model accounted for approximately 11% (*R*^2^=0.11) of the variation in EQ-5D index scores at follow-up, with the estimated regression coefficients and 95% CIs reported in Table 4. The model showed that EQ-5D index score at baseline, completion of the educational program, time spent on the app, and age were all significantly positively associated with follow-up EQ-5D index scores.

**Table 4:** Summary of multiple linear regression for EQ-5D index scores at follow-up.

Variables	Coefficient (95% CI)	*P* value
Baseline EQ-5D index score	0.214 (0.171-0.257)	<.001
Completion of educational program	0.061 (0.041-0.082)	<.001
Age	0.001 (0.000-0.002)	.02
Time spent on the app	0.0003 (0.0002-0.0004)	<.001

### Predictors of App Engagement (Time Spent on App)

Another stepwise multiple linear regression model was used to evaluate the predictor variables for time spent on the app with results reported in Table 5. The predictor variables evaluated in the model included baseline EQ-5D index scores, age, income, engagement with educational program, race and ethnicity, and sex. The best-fitting model accounted for approximately 4.1% (*R*^2^=0.041) of the variation in time spent on the app, with the estimated regression coefficients and 95% CIs reported in Table 5. The model showed that baseline EQ-5D index scores were significantly positively associated with app engagement, while incompletion of the educational program and Southeast Asian ethnicity were significantly negatively associated with app engagement.

**Table 5:** Summary of multiple linear regression for app engagement.

Variables	Coefficient (95% CI)	*P* value
Baseline EQ-5D index score	30.8 (11.1 to 50.5)	<.001
Incompletion of educational program	–35.5 (–45.3 to –25.6)	.002
Southeast Asian ethnicity	–21.4 (–40.5 to –2.32)	.03

## Discussion

### Overview

The growing burden of T2D continues to pose a serious public health risk with the increasing prevalence of the disease worldwide. It is recognized in the literature that, to a great extent, T2D arises due to the contribution of unhealthy lifestyle choices, such as poor diet and lack of physical activity, among other factors. Therefore, with no definitive cure for diabetes, self-management remains a vital component in the management of these patients.

The fast-developing nature of digital health applications means that these tools can be used to facilitate the self-management of many chronic conditions, including T2D. One of the fundamental goals of all diabetic management interventions is to improve quality of life. In this study, we used the self-reported EQ-5D-5L outcomes to assess the impact on HRQoL measures of participants following the 6-month use of the Gro Health app. Previous studies have supported the use of the EQ-5D-5L questionnaire as it is more discriminative than the EQ-5D-3L [26]. This study showed that engagement with the Gro Health digital app resulted in both statistically and clinically significant improvements in the self-reported quality of life outcomes among users with T2D and prediabetes. In a 2021 meta-analysis on the effectiveness of digital interventions on the self-management of patients with diabetes, no statistically significant changes in self-reported HRQoL were found [27]. Despite no statistical significance being noted in this study in any of the individual 5 domains of the EQ-5D when analyzed separately, the cumulative impact of combining these domains using the EQ-5D index scores revealed significant results at follow-up. The clinical significance of the EQ-5D index scores in this study was determined based on the findings reported by McClure et al [28] in adults with T2D, which showed that a change of at least 0.03 in the index score was significant. Additionally, the catalog of EQ-5D scores for the United Kingdom by Sullivan et al [29] discussed the loss of utility associated with T2D in the United Kingdom at 0.06, and our results showed that by follow-up, our cohort had a significant EQ-5D index score change of 0.05. This finding is supported by a 2016 review and a 2019 study by Jeffrey et al [30] confirming the benefits of mobile health apps in the care and self-management of patients with T2D [31].

This study showed 6% and 18% improvement from baseline in the EQ-5D index and VAS scores, respectively, among users. The mean baseline EQ-5D index scores for our cohort were 0.746 (SD 0.23) and the mean VAS score was 61.7 (SD 18.1), which were similar to findings reported by Grandy et al [32] among a population of patients with diabetes in the United Kingdom. Using the classification system used by Alshayban and Joseph [33] in a 2020 study of HRQoL among patients with T2D, we found that 66% (1164/1767) of our cohort had a moderate health state at baseline, with only 14% (250/1767) reporting severe health states. This could perhaps suggest that, contrary to the assumption that patients with more severe health states are more likely to engage with health apps, it is actually patients with a better health state who often engage with digital health tools. This was discussed by Birnbaum et al [34], where socioeconomic factors often associated with disease morbidity could be a potential barrier affecting patient engagement with digital health. This also raises the question of the responsiveness of the EQ-5D in populations that are not old or severely disabled, such as our cohort. In such cases, it is perhaps important to incorporate other condition-specific measures along with the EQ-5D as discussed by Payakachat et al [35] to improve the reliability of the HRQoL in the evaluation of interventions. Nevertheless, the reporting of the EQ-5D continues to remain valuable due to their role in measuring quality-adjusted life years, which is used in determining health economics and commissioning policies.

Several studies have reported lower HRQoL among female individuals with T2D compared to their male counterparts [36,37]. This study confirmed this, with female individuals having lower EQ-5D index scores at baseline compared to male individuals (Table 3). This finding could perhaps explain why significant improvements in EQ-5D index scores at follow-up were only noted among female individuals, since users with a lower EQ-5D index score are more likely to experience an improvement with time compared to those starting with a higher score at baseline. Users of the White race, along with those with an income of <£26,000 (US $33,000) were also noted to have positive improvements in EQ-5D index scores at follow-up compared to their counterparts. As highlighted earlier, this finding could be due to users with these ethnic and economic factors having a lower baseline EQ-5D index score compared to other groups in the subanalysis. In contrast, users of Southeast Asian ethnicity were found to have a lower EQ-5D index score at follow-up, despite an improvement in mean VAS score over the same period. This may be partially explained by the existing barriers to digital health access faced by patients from ethnic minority backgrounds, as described by Poduval et al [38] in their research, and the cultural appropriateness of the health advice could potentially affect its impact on users. Nevertheless, in this study, this subgroup of users was a minority of the sample, and therefore, this finding may not be adequately representative.

Despite the apparent impact of sex, race and ethnicity, and income on follow-up EQ-5D index scores, these variables were not identified as statistically significant in the regression analysis models. However, it is well established in the literature that socioeconomic and demographic factors are all known to contribute to HRQoL, and this would affect the EQ-5D index score at baseline, which was indeed found to be a positive predictor of EQ-5D index scores at follow-up. Furthermore, this study also identified age as a positive predictor variable of EQ-5D index scores at follow-up, although the effect of this was small and could have arisen due to the higher representation of younger users in the study sample, where more than half of the users (954/1767, 54%) were aged 50 years or younger. Engagement with the educational program and time spent in the app were also identified as significant positive predictors of follow-up EQ-5D index scores. This is consistent with findings by Kar et al [39] who also supported the effectiveness of digital educational interventions in the management of patients with T2D.

Engagement with the app was analyzed as time spent on the app, and this was positively associated with baseline EQ-5D index scores, although negatively associated with incompletion of the educational component and Southeast Asian ethnicity. This could be due to a lack of Southeast Asian language content while also consolidating the impression of existing ethnic inequalities in accessing digital health tools, which is similar to that described by a 2018 UK study [38]. Additionally, this finding could also explain why Southeast Asian users were the only group of users that were noted to have a lower EQ-5D index score at follow-up. Nonetheless, the Gro Health app is working to mitigate this by widening access through the development of more culturally sensitive features, including expanding the languages of the app.

There were several limitations to this study. Given the nature of the study as a service evaluation, there was no control group to assess changes in EQ-5D index scores in people with diabetes who did not use the Gro Health app. People who were more engaged could have opted to use the app as compared to those who are less likely to make positive changes to lifestyle, leading to a selection bias. The data were self-reported and collected in-app, with no measures to verify the accuracy of the data, potentially leading to information bias. Since this was a real-world data collection study, external factors such as changes in social circumstances, loss of employment, changes in physical health, or new medical comorbidities could have affected the index scores over the 6-month period that were not accounted for. Lastly, modeling of EQ-5D data is generally an unresponsive measure in populations that are not old or very disabled. As a result, no significant differences were identified in any of the 5 individual domains, despite overall improvements in the EQ-5D index and VAS scores during follow-up. Future areas of research should aim to assess the influencing factors of HRQoL in people with T2D in order to establish more sensitive outcome measures, either better or in addition to the EQ-5D, to assess the benefits of interventions on HRQoL. Additionally, a randomized clinical trial will be able to provide clear evidence while minimizing bias and confounding factors.

### Conclusions

Our findings demonstrate a significant positive effect on self-reported quality of life among people living with T2D and prediabetes engaging with a digital health intervention, Gro Health. This is likely facilitated through access to education, information provision, and monitoring support tools within the app. More efforts should be made to target ethnic minorities, who are known to have poor engagement with digital tools. Overall, this study contributes to the evidence supporting the incorporation of NHS-certified digital tools as an adjunct to the holistic management of people living with diabetes.

## Appendix

Multimedia Appendix 1 The GroHealth app.

Multimedia Appendix 2 EuroQoL EQ-5D-5L Questionnaire.

Multimedia Appendix 3 Summary of EQ-5D index scores according to baseline health status.

## Abbreviations

HRQoL: health-related quality of life
NHS: National Health Service
ORCHA: Organization for the Review of Care and Health Apps
T2D: type 2 diabetes
VAS: visual analogue scale
