# Supplementary figures and images for: Health Outcomes Following Engagement With a Digital Health Tool Among People With Prediabetes and Type 2 Diabetes: Prospective Evaluation Study
Source: JMIR Diabetes. 2023 Dec 28;8:e47224. doi: 10.2196/47224 (PMC10784975; doi:10.2196/47224)

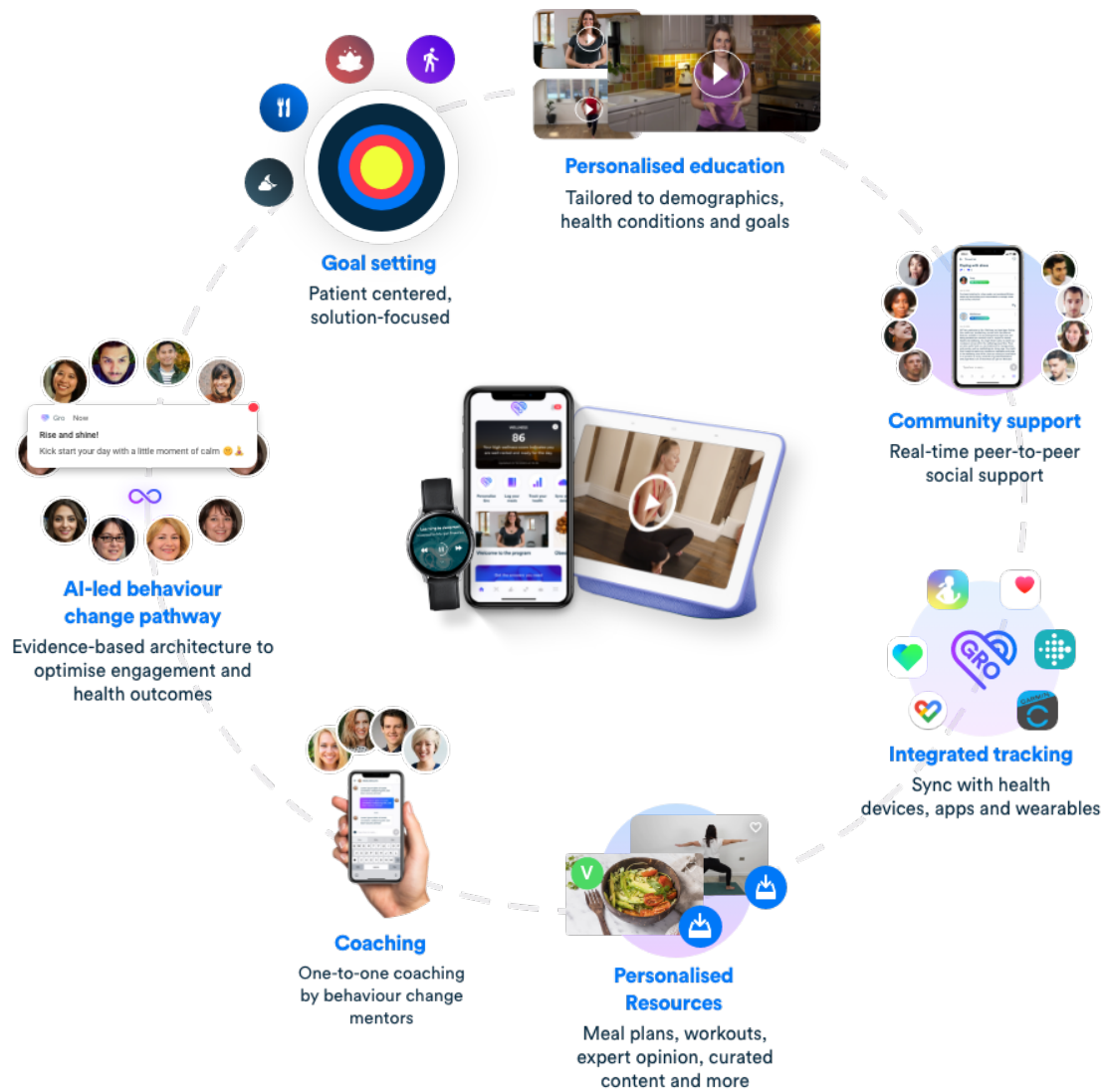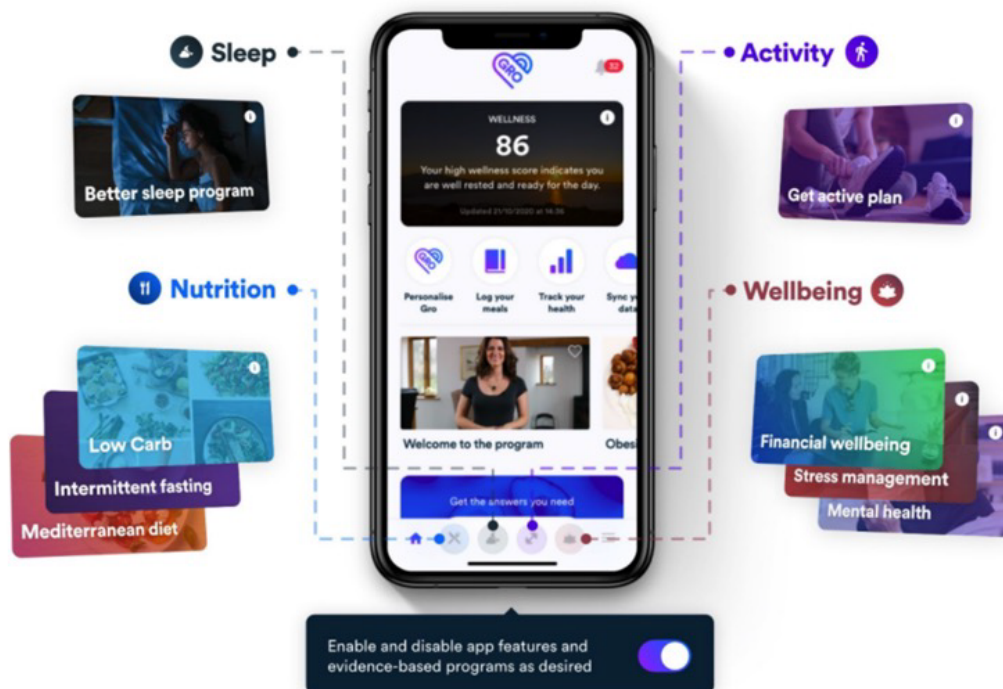

Supplement: Multimedia Appendix 1 [file diabetes_v8i1e47224_app1.pdf]
