# Supplementary material for: Health Outcomes Following Engagement With a Digital Health Tool Among People With Prediabetes and Type 2 Diabetes: Prospective Evaluation Study
Source: JMIR Diabetes. 2023 Dec 28;8:e47224. doi: 10.2196/47224 (PMC10784975; doi:10.2196/47224)
Supplement: Multimedia Appendix 3 [file diabetes_v8i1e47224_app3.pdf]

	Baseline EQ-5D index score, mean (SD)	Follow-up EQ-5D index score, mean (SD)
Perfect health	1.00 (0.00)	0.846 (0.20)
Moderate health	0.765 (0.09)	0.800 (0.21)
Severe health	0.298 (0.25)	0.680 (0.29)
